# Frailty and postoperative urinary tract infection

**DOI:** 10.1186/s12877-022-03461-1

**Published:** 2022-10-28

**Authors:** Susan A. Tuddenham, Susan L. Gearhart, E. James Wright III, Victoria L. Handa

**Affiliations:** 1 Department of Medicine, Johns Hopkins University School of Medicine, Baltimore, MD USA; 2 Department of Surgery, Johns Hopkins University School of Medicine, Baltimore, MD USA; 3 Department of Urology, Johns Hopkins University School of Medicine, Baltimore, MD USA; 4 Department of Gynecology and Obstetrics, Johns Hopkins University School of Medicine, Baltimore, MD USA

**Keywords:** Urinary tract infection, Frailty, National Surgical Quality Improvement Program

## Abstract

**Background:**

Among older adults, postoperative urinary tract infection is associated with significant harms including increased risk of hospital readmission and perioperative mortality. While risk of urinary tract infection is known to increase with age, the independent association between frailty and postoperative urinary tract infection is unknown. In this study we used 2014–2018 data from the U.S. National Surgical Quality Improvement Program (NSQIP) to investigate whether frailty is an independent risk factor for postoperative urinary tract infection, controlling for age and other relevant confounders.

**Methods:**

Frailty was assessed using the modified Frailty Index. Postoperative urinary tract infection was defined as any symptomatic urinary tract infection (of the kidneys, ureters, bladder, or urethra) developing within 30 days of the operative procedure. To examine associations between frailty and other specific factors and postoperative urinary tract infection, chi squared tests, students t-tests, and logistic regression modelling were used.

**Results:**

Urinary tract infection was identified after 22,356 of 1,724,042 procedures (1.3%). In a multivariable model controlling for age and other patient and surgical characteristics, the relative odds for urinary tract infection increased significantly with increasing frailty score. For example, compared to a frailty score of 0, the relative odds for urinary tract infection for a frailty score of 3 was 1.50 (95% confidence interval 1.41, 1.60). The relative odds associated with the maximum frailty score (5) was 2.50 (95% confidence interval 1.73, 3.61).

**Conclusions:**

Frailty is associated with postoperative urinary tract infection, independent of age. Further research should focus on the underlying mechanisms and strategies to mitigate this risk among frail adults.

**Supplementary Information:**

The online version contains supplementary material available at 10.1186/s12877-022-03461-1.

## Introduction

Urinary Tract Infections (UTIs) represent the most frequent infections in among adults over 60[1] and represent more than one-third of all health care acquired infections [2]. Postoperative UTI complicates 1–2% of surgical procedures in the US [3–5]. Postoperative UTIs are associated with increased postoperative morbidity, readmission risk and perioperative mortality [3, 6]. Given the significant harms of postoperative UTI in elderly patients, it is important to identify opportunities to reduce UTI incidence in this population.

Defining the relationship between frailty and postoperative UTI may represent an important step towards achieving this goal. Frailty is defined as a state of decreased physiologic reserve and resilience [7] and has emerged as a critical construct in geriatric medicine [8]. Frailty has been recognized as an important risk factor for multiple adverse medical outcomes [8], including perioperative morbidity, mortality, and readmission [3, 9–12]. Preoperative screening for frailty has increasingly been used to identify patients at high risk for poor perioperative outcomes and to target interventions to prevent complications [8].

Frailty has been associated with UTI in certain outpatient cohorts not specifically undergoing surgery [13, 14]. Frail adults may also plausibly be at increased risk to develop postoperative urinary tract infection [3, 11, 15], however the association between frailty and postoperative UTI may be confounded by other known risk factors for UTI, including advancing age, diabetes, malnutrition, and congestive heart failure[2, 16, 17]. Yet, to date, no dedicated studies have specifically focused on examining the relationship between frailty and postoperative UTI in elderly patients, while rigorously controlling for potential confounders.

The objective of this study is to investigate whether frailty is an independent risk factor for postoperative urinary tract infection, controlling for age and other relevant confounders. In this study, we utilized a large clinical database from the American College of Surgeons National Surgical Quality Improvement Program (NSQIP) to investigate associations between frailty and postoperative UTI in elderly patients, controlling for other known UTI risk factors. This question is important because results may support efforts to minimize perioperative risk for frail adults and to identify opportunities to improve the health of this vulnerable population.

## Methods

This study is an analysis of data provided by the American College of Surgeons NSQIP. This program includes > 800 hospitals nationwide. This outcomes-based program uses validated methods to collect perioperative data for the purpose of improving the quality of surgical care. NSQIP includes variables related to preoperative patient characteristics (age, gender, preoperative medical conditions), surgical classification (surgical specialty, inpatient versus outpatient surgery), and patient outcomes (postoperative morbidity, mortality, readmission). At each participating hospital, a trained and certified reviewer obtains relevant data elements using a variety of methods including medical chart abstraction. NSQIP data are de-identified and thus the Johns Hopkins Institutional Review Board deemed this research exempt (IRB00104750). This study utilized the 2014-8 NSQIP Participant Use Files as the data source.

Adults over the age of 60 years were considered in this analysis. The analysis included inpatient and outpatient surgery as well as both elective and emergent procedures. We excluded urologic and gynecologic surgical procedures, cases in which there was an active urinary tract infection prior to the principal operative procedure, and those with incomplete data.

The outcome for this analysis was postoperative UTI, described by the NSQIP study as any symptomatic urinary tract infection (of the kidneys, ureters, bladder, or urethra) that develops within 30 days of the principal operative procedure. The diagnosis of UTI requires at least one of the following symptoms: fever, urinary urgency, urinary frequency, dysuria, suprapubic tenderness, or costovertebral angle pain or tenderness. In the setting of at least one UTI symptom, this diagnosis is made if urine culture demonstrates > 100,000 colonies/ml.

The Modified Frailty Index (mFI-5)[18] was used to assess frailty in this population. The mFI-5 is a simplified version of the mFI-11 [12] and has been shown to predict postoperative complications in a variety of surgical subspecialties. The mFI-5 is based on preoperative functional status and the presence or absence of four medical conditions: diabetes, chronic obstructive pulmonary disease, congestive heart failure, and hypertension. Functional status is determined within 30 days prior to surgery and is defined as “independent” if the patient does not require any assistance from another person for any activities of daily living. The patient is considered “partially dependent” if he or she requires some assistance from another person for activities of daily living and “totally dependent” if he or she requires assistance for all activities of daily living. Using the five elements of the mFI-5, the resultant score ranges from 0 (no frailty) to 5 (most frail). The mFI-5 may be further dichotomized to “not frail” (0–1) versus “frail” (> 2). A score of > 2 corresponds to a “high” score on the original mFI-11 (≥ 4 of 11 frailty traits), which has been associated with a significant increase in the odds of serious postoperative complications [12].

We compared the characteristics of patients with and without UTI. Chi squared test was used for categorical data and Students t-test for continuous data. Demographic characteristics of interest included age and gender (male versus female). NSQIP data classified age in years until age 90; if age was greater than 90, it was classified as “90+” in the NSQIP dataset. For this analysis, we assigned an age of 95 years to those with age classified as “90+”. Each patient was also classified by preoperative functional status (independent versus partially or totally dependent), preoperative weight loss (> 10% body weight in the past 6 months), admission from a nursing home (or other chronic care facility), and elective versus urgent/ emergent surgery. Medical conditions of interest included diabetes, hypertension, congestive heart failure, chronic obstructive pulmonary disease, anemia (defined as preoperative hematocrit < 34%), disseminated cancer, and chronic steroid use. The relevant characteristics of the surgery included inpatient versus outpatient surgery and wound class (clean versus contaminated).

For each characteristic that differed significantly between patients with UTI and those without, we calculated the relative odds and 95% confidence interval utilizing logistic regression models. In a multivariable analysis, we considered the association between UTI and mFI-5 score, controlling for other relevant confounders. Stata/IC 16.1 (StatCorp LLC, College Station, TX) was used for all statistical analyses. We considered p < 0.05 significant in all analyses.

## Results

From 2014 to 2018, NSQIP data were available for 2,174,778 surgical cases among adults older than 60 years. Of these, 450,736 (20.7%) were excluded from this analysis. This included 6,634 cases with a preexisting UTI, 182,073 associated with urologic procedures, and 86,412 associated with gynecologic procedures. We also excluded 2 cases in which the sex of the patient was unknown, 1,921 cases that were not classified as either emergent or elective surgery, 11,671 for unknown preoperative functional status of the patient, and 192,023 without a preoperative hematocrit for identification of anemia. This analysis focused on the remaining 1,724,042 surgical cases.

In this population, postoperative UTI was identified in 22,356 cases (1.3%). A comparison of the cases with and without postoperative UTI (See Table1) suggested that patients who developed postoperative UTI were older, more likely to be female, more likely to have come to the hospital from a nursing home, more likely to have impaired functional status, and more likely to have medical co-morbidities. MFI-5 scores were also significantly associated with postoperative UTI (p < 0.001). The association with functional status was particularly notable: adults without functional independence were more than twice as likely to develop UTI (odds ratio 2.31, 95% confidence interval 2.21, 2.41). UTI was also associated with inpatient surgery, non-elective surgery, and “contaminated” or “clean-contaminated” surgery.

**Table 1:** Characteristics of the study population (n = 1,724,042), comparing patients who did or did not develop postoperative UTI.

	No postoperative UTI (n = 1,701,686)	Postoperative UTI (n = 22,356)	p
Age, years (mean ± SD)*	71.5 ± 8.4	74.8 ± 9.3	< 0.001
Frailty index score			< 0.001
0	469,709 (27.6%)	4,965 (22.2%)	
1	783,652 (46.1%)	9,813 (43.9%)	
2	381,880 (22.4%)	6,006 (26.9%)	
3	58,447 (3.4%)	1,320 (5.9%)	
4	7,329 (0.4%)	222 (1.0%)	
5	669 (< 0.1%)	30 (0.1%)	
Male gender	782,689 (46.0%)	7,977 (35.7%)	< 0.001
Diabetes	377,027 (22.2%)	5,661 (25.3%)	< 0.001
Hypertension	1,133,974 (66.6%)	15,691 (70.2%)	< 0.001
Congestive heart failure	276,062 (16.2%)	5,826 (26.1%)	< 0.001
Chronic obstructive pulmonary disease	131,430 (7.7%)	2,385 (10.7%)	< 0.001
Preoperative anemia	276,062 (16.2%)	5,826 (26.1%)	< 0.001
Disseminated cancer	52,061 (3.1%)	1,267 (5.7%)	< 0.001
Steroid use	79,416 (4.7%)	1,633 (7.3%)	< 0.001
Weight loss: >10% body weight in the past 6 months	30,348 (1.8%)	729 (3.3%)	< 0.001
Transferred from nursing home	31,898 (1.9%)	882 (4.0%)	< 0.001
Impaired preoperative functional status	84,613 (5.0%)	2,414 (10.8%)	< 0.001
Inpatient (vs. outpatient) surgery	1,274,941 (74.9%)	20,364 (91.1%)	< 0.001
Elective (vs. urgent or emergent) surgery	1,277,038 (75.1%)	13,415 (60.0%)	< 0.001
Wound “clean-contaminated” or “contaminated”	523,839 (30.8%)	8,856 (39.6%)	< 0.001

* In 51,263 cases, age was classified in NSQIP as “90+”. For this analysis, the age for individuals listed as “90+” was assumed to be 95 years.

In a multivariable model that included frailty score as an independent variable (Table2), the relative odds for UTI increased progressively with increasing mFI-5 score. Specifically, compared to an mFI-5 score of zero, a score of 1 was associated with a 6% increase in the odds for UTI (odds ratio 1.06, 95% confidence interval 1.03, 1.10). Similarly, a score of 3 was associated with a 50% increase in odds for UTI (odds ratio 1.50, 95% confidence interval 1.41, 1.60), and a score of 5 was associated with a 2.5 times increase in the relative odds for UTI (odds ratio 2.50, 95% confidence interval 1.73, 3.61). We found no significant collinearity (variance inflation factor approximately equal to 1.0) between any of the variables included in the model.

**Table 2:** Relative odds (and 95% confidence interval) for postoperative UTI (n = 1,724,042)

	Univariable OR	Multivariable OR with mFI-5*
mFI-5 score		
0	Reference	Reference
1	1.18 (1.14, 1.23)	1.06 (1.03, 1.10)
2	1.49 (1.43, 1.55)	1.27 (1.23, 1.32)
3	2.14 (2.01, 2.27)	1.50 (1.41, 1.60)
4	2.87 (2.50, 3.28)	1.82 (1.59, 2.09)
5	4.24 (2.94, 6.12)	2.50 (1.73, 3.61)
Age, per 10-year increase	1.52 (1.50, 1.54)	1.37 (1.34, 1.39)
Male gender	0.65 (0.63, 0.67)	0.67 (0.65, 0.69)
Diabetes	1.19 (1.16, 1.23)	†
Hypertension^†^	1.18 (1.15, 1.21)	†
Congestive heart failure^†^	1.83 (1.69, 1.98)	†
Chronic obstructive pulmonary disease^†^	1.43 (1.37, 1.49)	†
Preoperative anemia	1.82 (1.77, 1.88)	1.14 (1.10, 1.18)
Disseminated cancer	1.90 (1.80, 2.02)	1.56 (1.48, 1.66)
Steroid use	1.61 (1.53, 1.69)	1.42 (1.35, 1.49)
Weight loss: >10% body weight in the past 6 months	1.86 (1.72, 2.00)	1.26 (1.17, 1.36)
Transferred from nursing home	2.15 (2.01, 2.30)	1.09 (1.01, 1.17)
Impaired preoperative functional status^†^	2.31 (2.21, 2.41)	†
Inpatient (vs. outpatient) surgery	3.42 (3.27, 358)	2.63 (2.51, 2.76)
Elective (vs. urgent or emergent) surgery	0.50 (0.49, 0.51)	0.82 (0.79, 0.84)
Wound “clean-contaminated” or “contaminated”	1.48 (1.44, 1.51)	1.26 (1.23, 1.30)

* Modified frailty index

† These variables (diabetes, chronic obstructive pulmonary disease, congestive heart failure, treatment for hypertension, and impaired functional status) are included in the mFI-5 score and are therefore not included in a model that adjusts for mFI-5 score.

Approximately 3% of individuals had multiple surgeries during the index admission (though in the overall analysis we chose to analyze only the first or “index” surgery). However, because it would be difficult to reliably attribute the UTI to a specific surgery in these individuals, we conducted a sensitivity analysis excluding these cases (See **Supplemental Tables1 and 2**). In this analysis our findings were not substantially altered.

## Discussion

In this study of adults over the age of 60, we found that relative odds for postoperative UTI increased significantly and monotonically as frailty score increased, even after controlling for the confounding effect of other covariates (such as age). Of the five components of the mFI-5, the association between UTI and preoperative function status was particularly notable: the odds for UTI were more than doubled for adults who were partially or fully dependent on others for care. Results were similar after exclusion of patients with multiple surgeries during the index admission from analysis.

Our results do build on the findings of prior studies. In an analysis focused on the relationship between frailty and postoperative complications in patients of all ages undergoing lumbar fusion, Leven et al. [11] found an association between post-operative UTI and mFI-11 scores but this analysis did not control for relevant confounders, most notably age. Similarly Rothenberg et al.[3], found an association between frailty (evaluated with the Risk Analysis Index score) and postoperative UTI in univariate analysis in patients undergoing outpatient elective surgery. Finally, Amin et al. [15] found an association between frailty and postoperative complications (a composite variable including UTI, but not specifically assessing UTI alone), in patients undergoing urologic procedures. Our study is the first to focus specifically on postoperative UTIs in elderly patients across a wide variety of in and outpatient surgeries, and extends previous results to demonstrate that the association between frailty and postoperative UTI persists in a model that carefully controls for other relevant confounders, including age, gender, and medical comorbidities.

A strength of this research is that the sample size is quite large, providing the opportunity to consider multiple risk factors for UTI. Also, these data were derived from the NSQIP dataset and the process for data collection in NSQIP are highly rigorous; decreasing the likelihood of misclassification. However, there are a number of limitations as well. Data regarding the use of perioperative catheterization was not available. Catheterization is an important risk factor for UTI in hospitalized patients[2]. Thus, missing data for this variable may influence our results. For example, the use of an indwelling bladder catheter may confound some of these associations, such as the association between UTI and inpatient surgery. Additionally, data regarding perioperative antibiotics are not available. A prophylactic antibiotic at the time of surgery might affect perioperative UTI risk, especially in association with catheterization. The NSQIP includes a very wide variety of surgical procedures and therefore it would have been impossible to adjust for each type of surgery, although we were able to adjust for certain surgical characteristics, including elective versus urgent, in versus outpatient and “clean contaminated” versus “contaminated” wound type. Although we defined frailty using the mFI-5, this is only one of several validated frailty measures [8]. Frailty indices, like the mFI-5, are based on the presence or absence of comorbid conditions, sometimes incorporating other factors such as social and cognitive conditions. In contrast, frailty phenotypic measures (such as the Edmonton frailty score [19]) require an assessment of motor function and functional abilities. There is no consensus on the best classification system for measuring frailty in research or clinical practice. We did consider using the RAI (Risk Analysis Index) score which has been employed in on some other studies [3], however this index requires an assessment of cognition, which was missing for 16% of cases in our dataset, whereas the mFI-5 could be calculated for all but 1% of cases. Agreement between various frailty measures has been variable [20] and thus our results may not pertain to other frailty measures.

Our findings, showing an independent association between frailty and postoperative UTI, suggest multiple avenues for future research. First, clinical trials among elderly patients could assess the value of prevention strategies for reducing postoperative UTI in those frail patients identified to be at particularly high risk by our study. Prevention strategies might involve systems-level operational changes, such as interventions aimed at shortening urinary catheterization time and multimodal pain control regimens that are less likely than opioids to impact mobility or to depress immune function [21, 22]. New therapeutic modalities may be an option as well. For example, antibiotic-sparing interventions to prevent recurrent UTI in the ambulatory setting may be of value in the perioperative setting (such as D-mannose [23], methenamine [24], or intravaginal probiotics for female patients [25]). Importantly, we found that frailty, even when controlling for age and a host of other confounders and comorbidities, is associated with the outcome, showing that this simple frailty risk score may be a practical tool to use to identify those at highest risk of post-operative UTI, who could be targeted by existing or novel UTI risk-mitigating interventions.

Additionally, our findings raise important questions about the mechanism for the observed association between frailty and UTI. It is important to note that frailty has been associated with recurrent UTI in ambulatory urologic practice[14], and in outpatients with a history of diabetes and chronic kidney disease[13], suggesting that frailty may put adults at risk for UTI in other settings. Possible mechanisms for an association between frailty and UTI include worse perineal hygiene among frail adults, possible alterations in the microbiome, or impaired immune response in these individuals. Notably, frailty has been linked to immune system dysregulation and a pro-inflammatory phenotype. What mediates these findings is unclear, but significant interest has developed regarding the role of the gut microbiota in driving inflammation in frail adults [26]. In the future, microbiota-based interventions such as oral probiotics could offer ways to improve immunity and decrease inflammation in frail patients [25], perhaps including those susceptible to UTIs.

## Conclusion

In this study, we found that relative odds for postoperative UTI increased significantly as frailty score increased, with those with the highest frailty score having over twice the odds of having a postoperative UTI. Future studies should consider targeting interventions for UTI prevention in these highest risk individuals.

## Electronic supplementary material

Below is the link to the electronic supplementary material.

Supplementary Material 1.
Supplementary Table 1: Characteristics of the study population (n=1,660,993), comparing patients who did or did not develop postoperative UTI, excluding those with multiple surgeries during the index admission
Supplementary Table 2: Relative odds (and 95% confidence interval) for postoperative UTI (n=1,660,993) excluding those with multiple surgeries during the index admission

## Data Availability

The data that support the findings of this study are available from the American College of Surgeons NSQIP program but restrictions apply to the availability of these data, which were used under license for the current study, and so are not publicly available. Data are however available from the authors upon reasonable request and with permission of the American College of Surgeons.

## Abbreviations

UTI: Urinary Tract Infection.

## References

[1] Nicolle LE (2002). Urinary tract infection in geriatric and institutionalized patients. Curr Opin Urol.

[2] Ramanathan R, Duane TM (2014). Urinary tract infections in surgical patients. Surg Clin North Am.

[3] Rothenberg KA, Stern JR, George EL, Trickey AW, Morris AM, Hall DE, Johanning JM, Hawn MT, Arya S (2019). Association of Frailty and Postoperative Complications With Unplanned Readmissions After Elective Outpatient Surgery. JAMA Netw Open.

[4] Bucher BT, Ferraro JP, Finlayson SRG, Chapman WW, Gundlapalli AV (2019). Use of Computerized Provider Order Entry Events for Postoperative Complication Surveillance. JAMA Surg.

[5] Alvarez AP, Demzik AL, Alvi HM, Hardt KD, Manning DW (2016). Risk Factors for Postoperative Urinary Tract Infections in Patients Undergoing Total Joint Arthroplasty. Adv Orthop.

[6] Lisk R, Uddin M, Parbhoo A, Yeong K, Fluck D, Sharma P, Lean MEJ, Han TS (2019). Predictive model of length of stay in hospital among older patients. Aging Clin Exp Res.

[7] Fried LP, Tangen CM, Walston J, Newman AB, Hirsch C, Gottdiener J, Seeman T, Tracy R, Kop WJ, Burke G (2001). Frailty in older adults: evidence for a phenotype. J Gerontol A Biol Sci Med Sci.

[8] Walston J, Buta B, Xue QL (2018). Frailty Screening and Interventions: Considerations for Clinical Practice. Clin Geriatr Med.

[9] Robinson TN, Walston JD, Brummel NE, Deiner S, Brown CHt, Kennedy M, Hurria A: Frailty for Surgeons: Review of a National Institute on Aging Conference on Frailty for Specialists. J Am Coll Surg 2015, 221(6):1083–1092. 10.1016/j.jamcollsurg.2015.08.428 PMC4673051 26422746

[10] Shinall MC, Arya S, Youk A, Varley P, Shah R, Massarweh NN, Shireman PK, Johanning JM, Brown AJ, Christie NA (2020). Association of Preoperative Patient Frailty and Operative Stress With Postoperative Mortality. JAMA Surg.

[11] Leven DM, Lee NJ, Kim JS, Kothari P, Steinberger J, Guzman J, Skovrlj B, Shin JI, Phan K, Caridi JM (2017). Frailty Is Predictive of Adverse Postoperative Events in Patients Undergoing Lumbar Fusion. Global Spine J.

[12] Seib CD, Rochefort H, Chomsky-Higgins K, Gosnell JE, Suh I, Shen WT, Duh QY, Finlayson E (2018). Association of Patient Frailty With Increased Morbidity After Common Ambulatory General Surgery Operations. JAMA Surg.

[13] Chao CT, Lee SY, Wang J, Chien KL, Huang JW (2021). Frailty increases the risk for developing urinary tract infection among 79,887 patients with diabetic mellitus and chronic kidney disease. BMC Geriatr.

[14] Tang M, Quanstrom K, Jin C, Suskind AM (2019). Recurrent Urinary Tract Infections are Associated With Frailty in Older Adults. Urology.

[15] Amin KA, Lee UJ, Jin C, Boscardin J, Medendorp AR, Anger JT, Suskind AM (2019). "A National Study Demonstrating the Need for Improved Frailty Indices for Preoperative Risk Assessment of Common Urologic Procedures". Urology.

[16] Kang CY, Chaudhry OO, Halabi WJ, Nguyen V, Carmichael JC, Mills S, Stamos MJ (2012). Risk factors for postoperative urinary tract infection and urinary retention in patients undergoing surgery for colorectal cancer. Am Surg.

[17] Soriano A, Hassani D, Harvie H, Sheyn D (2021). Incidence of and Risk Factors for Postoperative Urinary Tract Infection After Abdominal and Vaginal Colpopexy. Female Pelvic Med Reconstr Surg.

[18] Subramaniam S, Aalberg JJ, Soriano RP, Divino CM (2018). New 5-Factor Modified Frailty Index Using American College of Surgeons NSQIP Data. J Am Coll Surg.

[19] Rolfson DB, Majumdar SR, Tsuyuki RT, Tahir A, Rockwood K (2006). Validity and reliability of the Edmonton Frail Scale. Age Ageing.

[20] Aguayo GA, Donneau AF, Vaillant MT, Schritz A, Franco OH, Stranges S, Malisoux L, Guillaume M, Witte DR (2017). Agreement Between 35 Published Frailty Scores in the General Population. Am J Epidemiol.

[21] Wiese AD, Griffin MR, Schaffner W, Stein CM, Greevy RA, Mitchel EF, Grijalva CG (2019). Long-acting Opioid Use and the Risk of Serious Infections: A Retrospective Cohort Study. Clin Infect Dis.

[22] Stephan F, Sax H, Wachsmuth M, Hoffmeyer P, Clergue F, Pittet D (2006). Reduction of urinary tract infection and antibiotic use after surgery: a controlled, prospective, before-after intervention study. Clin Infect Dis.

[23] Lenger SM, Bradley MS, Thomas DA, Bertolet MH, Lowder JL, Sutcliffe S (2020). D-mannose vs other agents for recurrent urinary tract infection prevention in adult women: a systematic review and meta-analysis. Am J Obstet Gynecol.

[24] Forbes R, Ali A, Abouhajar A, Brennand C, Brown H, Carnell S, Chadwick T, Eardley I, Lecouturier J, Mossop H (2018). ALternatives To prophylactic Antibiotics for the treatment of Recurrent urinary tract infection in women (ALTAR): study protocol for a multicentre, pragmatic, patient-randomised, non-inferiority trial. Trials.

[25] Stapleton AE, Au-Yeung M, Hooton TM, Fredricks DN, Roberts PL, Czaja CA, Yarova-Yarovaya Y, Fiedler T, Cox M, Stamm WE (2011). Randomized, placebo-controlled phase 2 trial of a Lactobacillus crispatus probiotic given intravaginally for prevention of recurrent urinary tract infection. Clin Infect Dis.

[26] Piggott DA, Tuddenham S (2020). The gut microbiome and frailty. Transl Res.

