# Supplementary material for: Frailty and postoperative urinary tract infection
Source: BMC Geriatr. 2022 Oct 28;22:828. doi: 10.1186/s12877-022-03461-1 (PMC9617308; doi:10.1186/s12877-022-03461-1)
Supplement: Supplementary file 1 — Supplementary Material 1. Supplementary Table 1: Characteristics of the study population (n=1,660,993), comparing patients who did or did not develop postoperative UTI, excluding those with multiple surgeries during the index admission Supplementary Table 2: Relative odds (and 95% confidence interval) for postoperative UTI (n=1,660,993) excluding those with multiple surgeries during the index admission [file 12877_2022_3461_MOESM1_ESM.docx]

| Supplementary Table 1: Characteristics of the study population (n=1,660,993), comparing patients who did or did not develop postoperative UTI, excluding those with multiple surgeries during the index admission | | | |
| --- | --- | --- | --- |
|  | No postoperative UTI  (n=1,640,739) | Postoperative UTI  (n=20,254) | p |
| Age, years (mean ± SD)* | 71.5 ± 8.4 | 75.0 ± 9.3 | <.001 |
| Frailty index score |  |  | <.001 |
| 0 | 457,145 (27.9%) | 4,570 (22.6%) |  |
| 1 | 757,236 (46.2%) | 8,951 (44.2%) |  |
| 2 | 364,671 (22.2%) | 5,363 (26.5%) |  |
| 3 | 54,401 (3.0%) | 1,150 (5.7%) |  |
| 4 | 6,677 (0.4%) | 193 (1.0%) |  |
| 5 | 609 (<0.1%) | 27 (0.1%) |  |
| Male gender | 749,950 (45.7%) | 7,118 (35.1%) | <.001 |
| Diabetes | 360,017 (21.9%) | 5,033 (24.9%) | <.001 |
| Hypertension | 1,090,169 (66.4%) | 14,155 (69.9%) | <.001 |
| Congestive heart failure | 26,244 (1.6%) | 597 (3.0%) | <.001 |
| Chronic obstructive pulmonary disease | 123,855 (7.6%) | 2,201 (10.4%) | <.001 |
| Preoperative anemia | 258,766 (15.8%) | 5,160 (25.5%) | <.001 |
| Disseminated cancer | 49,264 (3.0%) | 1,137 (5.6%) | <.001 |
| Steroid use | 75,165 (4.6%) | 1,434 (7.1%) | <.001 |
| Weight loss: >10% body weight in the past 6 months | 28,108 (1.7%) | 641 (3.2%) | <.001 |
| Transferred from nursing home | 30,001 (1.8%) | 788 (3.9%) | <.001 |
| Impaired preoperative functional status | 79,249 (4.8%) | 2,148 (10.6%) | <.001 |
| Inpatient (vs outpatient) surgery | 1,219,997 (74.4%) | 18,346 (90.6%) | <.001 |
| Elective (vs urgent or emergent) surgery | 1,241,411 (75.7%) | 12,284 (60.7%) | <.001 |
| Wound “clean-contaminated” or “contaminated” | 497,970 (30.4%) | 7,907 (39.0%) | <.001 |
| * In 47,088 cases, age was classified in NSQIP as “90+”. For this analysis, the age for individuals listed as “90+” was assumed to be 95 years. | | | |

| Supplementary Table 2: Relative odds (and 95% confidence interval) for postoperative UTI (n=1,660,993) excluding those with multiple surgeries during the index admission | | |
| --- | --- | --- |
|  | Univariable OR | Multivariable OR with mFI-5* |
| mFI-5 score  0  1  2  3  4  5 | Reference  1.18 (1.14, 1.23)  1.47 (1.41, 1.53)  2.11 (1.98, 2.26)  2.89 (2.50, 3.35)  4.44 (3.01, 6.53) | Reference  1.06 (1.02, 1.09)  1.26 (1.21, 1.31)  1.48 (1.38, 1/58)  1.83 (1.58, 2.12)  2.60 (1.76, 3.83) |
| Age, per 10-year increase | 1.55 (1.52, 1.57) | 1.39 (1.37, 1.41) |
| Male gender | 0.64 (0.63, 0.66) | 0.66 (0.64, 0.68) |
| Diabetes | 1.18 (1.14, 1.21) | † |
| Hypertension^†^ | 1.17 (1.14, 1.21) | † |
| Congestive heart failure^†^ | 1.87 (1.72, 2.03) | † |
| Chronic obstructive pulmonary disease^†^ | 1.42 (1.35, 1.48) | † |
| Preoperative anemia | 1.83 (1.77, 1.88) | 1.13 (1.09, 1.17) |
| Disseminated cancer | 1.92 (1.81, 2.04) | 1.58 (1.48, 1.68) |
| Steroid use | 1.59 (1.50, 1.68) | 1.41 (1.33, 1.49) |
| Weight loss: >10% body weight in the past 6 months | 1.88 (1.73, 2.03) | 1.27 (1.17, 1.38) |
| Transferred from nursing home | 2.17 (2.02, 2.24) | 1.08 (1.00, 1.16) |
| Impaired preoperative functional status^†^ | 2.34 (2.23, 2.45) | † |
| Inpatient (vs outpatient) surgery | 3.31 (3.16, 3.48) | 2.55 (2.43, 2.68) |
| Elective (vs urgent or emergent) surgery | 0.50 (0.48, 0.51) | 0.82 (0.79, 0.85) |
| Wound “clean-contaminated” or “contaminated” | 1.47 (1.43, 1.51) | 1.26 (1.23, 1.30) |
| * Modified frailty index  † These variables (diabetes, chronic obstructive pulmonary disease, congestive heart failure, treatment for hypertension, and impaired functional status) are included in the mFI-5 score and are therefore not included in a model that adjusts for mFI-5 score. | | |
